# Heterozygous loss of keratinocyte TRIM16 expression increases melanocytic cell lesions and lymph node metastasis

**DOI:** 10.1007/s00432-019-02981-5

**Published:** 2019-07-24

**Authors:** Selina K. Sutton, Belamy B. Cheung, Hassina Massudi, Owen Tan, Jessica Koach, Chelsea Mayoh, Daniel R. Carter, Glenn M. Marshall

**Affiliations:** 1 Children’s Cancer Institute Australia for Medical Research, Lowy Cancer Research Centre, UNSW Sydney, Kensington, NSW Australia; 2 School of Women’s and Children’s Health, UNSW Sydney, Randwick, NSW 2031 Australia; 3 School of Biomedical Engineering, University of Technology Sydney, Ultimo, Australia; 4 Kids Cancer Centre, Sydney Children’s Hospital, Randwick, NSW 2031 Australia

**Keywords:** Melanoma, TRIM16, Keratinocyte, Carcinogenesis, Migration, Metastasis

## Abstract

**Purpose:**

The tripartite motif (TRIM)16 acts as a tumour suppressor in both squamous cell carcinoma (SCC) and melanoma. TRIM16 is known to be secreted by keratinocytes, but no studies have been reported yet to assess the relationship between TRIM16 keratinocyte expression and melanoma development.

**Methods:**

To study the role of TRIM16 in skin cancer development, we developed a keratinocyte TRIM16-specific knockout mouse model, and used the classical two-stage skin carcinogenesis challenge method, to assess the loss of keratinocyte TRIM16 on both papilloma, SCC and melanoma development in the skin after topical carcinogen treatment.

**Results:**

Heterozygous, but not homozygous, TRIM16 knockout mice exhibited an accelerated development of skin papillomas and melanomas, larger melanoma lesions and an increased potential for lymph node metastasis.

**Conclusion:**

This study provides the first evidence that keratinocyte loss of the putative melanoma tumour suppressor protein, TRIM16, enhances melanomagenesis. Our data also suggest that TRIM16 expression in keratinocytes is involved in cross talk between keratinocytes and melanocytes, and has a role in melanoma tumorigenesis.

## Introduction

The tumour suppressive and tumour promoting effects of proteins secreted by keratinocytes on melanoma development have been previously reported. These include the increased expression and paracrine secretion of maspin by keratinocytes during senescence that inhibits endothelial cell angiogenesis (Nickoloff et al. 2004) and the keratinocyte secretion of matrix metalloproteinase-9 in facilitating melanoma invasion in a reconstructed skin model (Van Kilsdonk et al. 2010). Genetically engineered mouse (GEM) models are also used to determine loss of function (knockout, knockdown or dominant-negative) or gain of function (knock-in, transgenic) (Cheon and Orsulic 2011). There is also scope to cross GEM mice with other tumour suppressor knockout mice and determine tumour development if a single gene knockout is insufficient to develop tumours alone, or with carcinogen challenge (Dankort et al. 2009; Sotillo et al. 2001). One mouse model system that established CDK4 as a melanoma oncogene used a knock-in of CDK4, which alone was not sufficient to induce tumorigenesis, but required carcinogen challenge before the phenotype was revealed (Sotillo et al. 2001). Mice that harbour a conditional melanocyte-specific expression of oncoprotein BRAFV600E develop melanocytic hyperplasia that is benign due to the induction of senescence (Dankort et al. 2009; Dhomen et al. 2009). Combination of BRAFV600E with PTEN tumour suppressor gene silencing resulted in the development of melanomas with 100% penetrance.

The tripartite motif-containing (TRIM) proteins family is functionally diverse and involved in cellular processes including cell cycle regulation, proliferation, differentiation, ubiquitination, apoptosis, tumour suppress functions and oncogenesis(Sardiello et al. 2008). One of TRIM protein, TRIM16, has been demonstrated to acts as a tumour suppressor protein in neuroblastoma via effects on cytoplasmic vimentin and nuclear E2F1 (Marshall et al. 2010). TRIM16 binds vimentin in the cytoplasm and causes a reduction in vimentin protein expression. Conversely, gene silencing of TRIM16 which is mediated by siRNA results in an increase in vimentin protein expression. Further, the downregulation of vimentin is required for the ability of TRIM16 to inhibit neuroblastoma cell migration (Marshall et al. 2010). Additional work has demonstrated that TRIM16 inhibits neuroblastoma cell proliferation through cell cycle regulation and localization to the nucleus (Bell et al. 2013). High nuclear staining of TRIM16 has been observed in differentiating ganglia cells which is absent in the tumour initiating cells. TRIM16 protein translocates to the nucleus in the G1 cell cycle phase after being upregulated. Cell cycle progression is attenuated through changes in cyclin D1 and p27. These data implicate TRIM16 as a regulator of G1/S progression and cellular differentiation (Bell et al. 2013). TRIM16 translocates to the nucleus upon treatment with all-trans retinoic acid and binds to and downregulates E2F1 protein, reducing E2F1 protein half-life (Marshall et al. 2010). TRIM16 has further been shown to induce apoptosis in neuroblastoma cells (BE-(2)-C) and breast cancer cells (MCF-7) in a caspase-2-dependent manner (Kim et al. 2013). In addition, TRIM16 has been shown to restore retinoid sensitivity to retinoid-resistant breast and lung cancer cells via epigenetic mechanism of histone acetylation and restoration of RARβ2 transcription (Raif et al. 2009).

Previously, we have also shown that the putative tumour suppressor, TRIM16, is upregulated during keratinocyte differentiation, and TRIM16 protein expression is lost during the progression of normal skin through SCC development (Cheung et al. 2012). Furthermore, TRIM16 is lost during the progression of melanoma and correlates with human melanoma metastasis (Sutton et al. 2014). TRIM16 protein has been shown to be actively secreted by keratinocytes in response to UV light in a caspase-1-dependent manner (Munding et al. 2006a; Feldmeyer et al. 2007). TRIM16 binds to components of the inflammasome complex and pro-IL1β, which is associated with innate immune response in keratinocytes. These reports suggest that TRIM16 has functional roles in skin physiology and pathology, and, in immune responses. TRIM16 is known to be secreted by keratinocytes; however, the effect of the loss of keratinocyte TRIM16 on skin cancer development is currently unknown. Here, we investigated the effect of reduced TRIM16 expression in keratinocytes on carcinogen-induced malignancy of the skin.

## Materials and methods

### Design of the TRIM16 knockout construct

TRIM16 knockout mice were generated at Ozgene Australia, utilizing a construct designed to ablate the full-length TRIM16 coding sequence flanked by lox P sites. We crossed TRIM16 wild-type/flox knockout (KO) mice with skin-specific Cre mice, which express Cre under the control of the human keratin 14 promoter, to allow excision of full-length TRIM16 and Neo selection cassette in tissues expressing keratin-14. The heterozygous and homozygous of skin specific TRIM16 KO mice are viable and fertile.

### Preparation of TRIM16 floxed mice

For modelling of the function of TRIM16 in melanomagenesis, we chose a LoxP–Cre knockout system that involves knocking in a LoxP–Cre construct with flanking homology to the TRIM16 gene resulting in a ‘floxed’ mouse and gene deletion by crossing with a Cre expression system under the control of a promoter for the tissue of interest. The skin-specific knockout mouse model was produced from crossing our floxed mice with a Cre delete mouse under the control of Keratin 14. Keratin 14 is expressed in mitotically active basal layer cells and its expression is downregulated as cells differentiate (Alam et al. 2011a). Knocking out TRIM16 in keratin 14 expressing cells ensures a specific knockout of TRIM16 in the epithelial cells (Alam et al. 2011b; Hafner et al. 2004) while the remaining mouse tissues express TRIM16. Knocked-out tissues include the skin, tongue and cornea epithelial cells (Alam et al. 2011a).

### Genotyping PCR design to determine TRIM16 knockout

Genotyping of the floxed mice required the recognition of the Neo sequence that is present in the knocked-in flox construct. Amplification of the Neo cassette confirms at least one copy of the flox construct, while amplification of Exon 6 of the wild-type TRIM16 (spanning an intronic sequence), confirms the presence of wild-type TRIM16. Combination of these two primer pairs in a multiplex assay allows the determination of the three genotypes where wild-type mice will only amplify the Exon 6 PCR, homozygous floxed mice will only amplify the neo cassette and heterozygous floxed mice will amplify for both Exon 6 and the Neo cassette. For the skin-specific TRIM16 knockout mice, a KRT14Cre delete strain is used to excise the flox construct and remove the TRIM16 gene. As this Cre delete strain is under the control of a KRT14 promoter, only the keratinocytes expressing Keratin 14 will be knocked out for the TRIM16 gene. To determine if the deletion of the TRIM16 gene is efficient, a knockout (KO) PCR is used, which is designed to amplify spanning the region where the TRIM16 was before excision. Confirmation of the presence of the TRIM16 gene is determined by amplification of the first exon (Exon 1). Thus, combination of the KO PCR and Exon 1 PCR distinguishes between the presence of all three genotypes. Genotyping of mice was performed using DNA extracted by Chelex (Sigma-Aldrich, NSW, Australia) from tail tips using the following primer pairs (Table 1).

**Table 1:** Primer sequences for genotyping TRIM16 knockout mice

PCR	Directions	Sequences 5′–3′
Neo	Forward	AGAGGCTATTCGGCTATGACTGG
	Reverse	GGACAGGTCGGTCTTGACAAAAAG
Exon 6	Forward	TGCCTTGTGGGGGTCACTTGGA
	Reverse	GGTGTTCCCAGGGCGTGGTG
KO PCR	Forward	GAGCCTCGTCCTGTCTGAGTAAC
	Reverse	AAACCAAGAAGTGCCAGAAATA
Exon 1	Forward	GAGCCTCGTCCTGTCTGAGTAAC
	Reverse	TCTTCTTTTTCTGCTGGGATAG
β2 microglobulin	Forward	TCTCACTGACCGGCCTGTAT
	Reverse	GGAACTGTGTTACGTAGCAG

### Development of skin lesions using a two-stage skin carcinogenesis model

TRIM16 wild-type, heterozygous and homozygous skin-specific littermate mice between 7 and 9 weeks of age were shaved on the right dorsal flank prior to initiation. Mice were initiated with a single topical dose of 7,12-dimethylbenz(a)anthracene (DMBA) at 97.5 nmol in 0.2 mL of acetone. 2 weeks following initiation, mice were treated with either 0.2 mL 6.8 nmol of 12-O-tetradecanoylphorbol-13-acetate (TPA) or acetone control twice weekly for a period of 21 weeks. Mice were scored for skin lesion development weekly throughout the study.

### Culture of primary keratinocytes

Primary keratinocytes were cultured from the tails of 7-week-old mice. Mice were euthanized with CO_2_, and then the tail was removed and placed in 80% ethanol. Tail skin was removed with sterile dissecting tools and cut into 1 cm sections and washed in epidermal keratinocyte culture media (CnT) (CellnTec, Bern, Switzerland). Sections were cultured overnight in CnT media with 5 mg/mL dispase and penicillin/streptomycin antibiotic. After rubbing tissue to release a single cell suspension, the tissue was centrifuged and the resulting pellet was cultured in CnT media with antibiotics in collagen IV (BD Biosciences, NSW, Australia) coated dishes. Western blotting for TRIM16 protein was performed from cultured keratinocytes using a TRIM16-specific antibody. Gene expression of TRIM16 was performed by RT-PCR using TRIM16 primers forward 5′-GGCTCTCTGGTTTGACTTGG-3′, reverse 5′-GGTTTCTTCGGTGGAAAACAA- 3′ and β2 M primers forward 5′-TCTCACTGACCGGCCTGTAT-3′, reverse 5′-GGAACTGTGTTACGTAGCAG-3′ in PCR multiplex.

### Immunofluorescence

Five-micrometre-thick tissue sections of lymph nodes were cut from the paraffin blocks and placed on silane-coated slides. Slides were dewaxed in two exchanges of xylene and rehydrated using an ethanol gradient. Non-specific antigens were neutralized with 10% FCS for 1 h at room temperature. Slides were probed with Melan-A antibody (Santa Cruz) at 1:200 dilution. The secondary antibody (1/500) Alexa fluor 488 anti-mouse or Alexa fluor 594 anti-mouse (Abcam) was applied in 10%FCS/PBS for 1 h. Mounting media with DAPI (Life technologies, NSW, Australia) were applied and the coverslip was secured before viewing on the Olympus Fluoview FV1000 fluorescent microscope.

### Statistical analysis

Statistical analysis for all data was performed using GraphPad Prism version 6.0 (GraphPad software, La Jolla, CA, USA). ANOVA statistical analysis was applied to papilloma and lesion development between genotypes over a time course. The Student’s *t* test was applied to all other statistical analysis.

## Results

### Characterization of TRIM16 skin-specific knockout mice

**Fig. 1:**
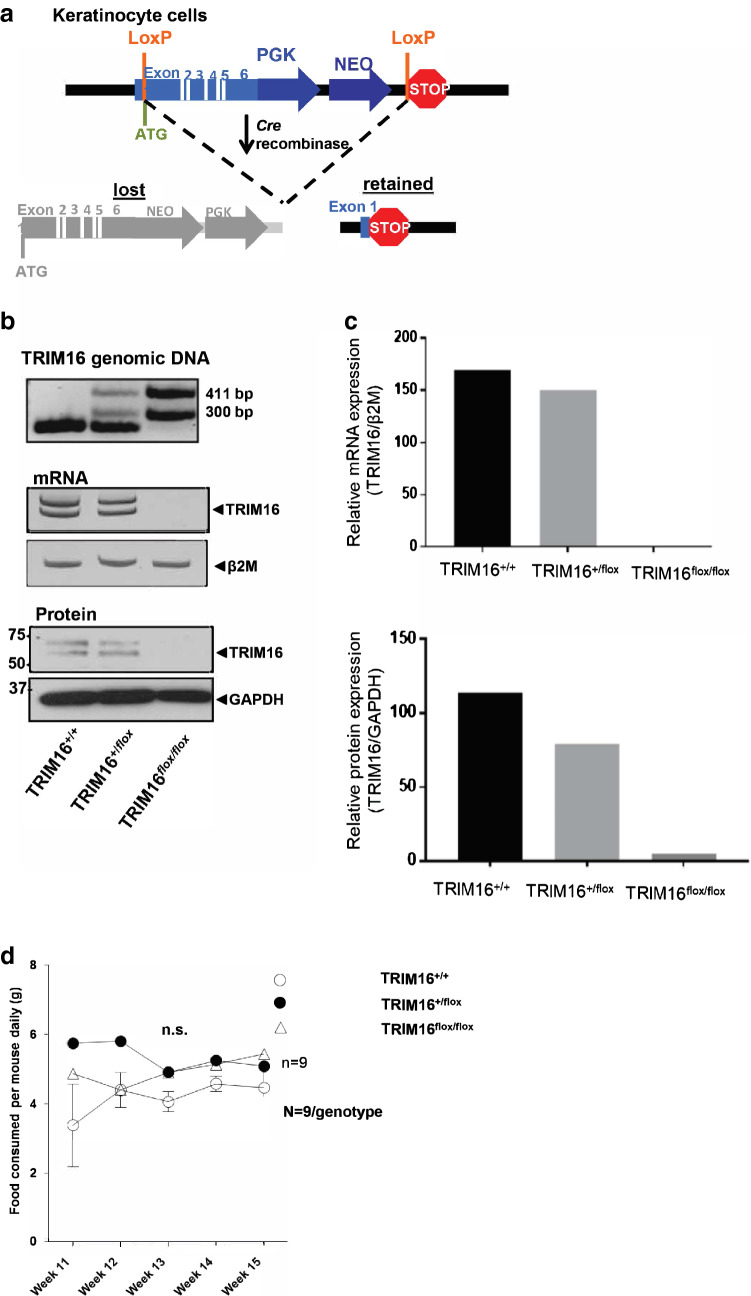
Characterization of TRIM16 skin-specific knockout mice. **a** Design of the floxed TRIM16 mouse model, indicating the LoxP sites (orange) flanking Exon 1–6 of the mouse TRIM16 gene. Deleted and retained regions are shown after Cre deletion. **b** Targeted disruption of the TRIM16 gene, is confirmed by PCR for TRIM16 genomic DNA and mRNA, and immunoblot for mouse TRIM16 protein of keratinocyte primary culture from TRIM16 wild-type (TRIM16^+/+^), TRIM16 heterozygous (TRIM16^+/flox^) and TRIM16 homozygous (TRIM16^flox/flox^) mice. **c** Densitometry quantification of TRIM16 mRNA and protein expression in keratinocyte cultures from wild-type, heterozygous, and homozygous mice. **d** TRIM16 keratinocytes-specific knockout mice TRIM16^+/+^ wild-type, TRIM16^+/flox^ heterozygous and TRIM16^flox/flox^ homozygous mice (*N* = 9/genotype) were monitored for food consumption/mouse for 5 weeks. Two-way ANOVA was performed to determine statistical significance

To understand the role of TRIM16 in cancer development, we developed a keratinocyte-specific TRIM16 knockout mouse model. Our design of the TRIM16 keratinocyte knockout model used the insertion of LoxP sites into the TRIM16 gene spanning exons 1–6 in preparation for Cre deletion (Fig. 1a). As we aimed to produce a keratinocyte-specific knockout model, we selected the KRT14-*Cre* mouse for deletion crossing (Fig. 1a) leaving other tissues unaffected. TRIM16 skin-specific knockout mice are viable, and expected Mendelian birth ratios are observed. Confirmation of the successful deletion of the TRIM16 gene was evidenced by PCR using genomic DNA and RT-PCR for mRNA expression, showing slightly reduced mRNA expression in heterozygous TRIM16^+/flox^ mouse, and the absence of the TRIM16 a and b isoforms in the homozygous TRIM16^flox/flox^ mouse by RT-PCR of mRNA from cultured keratinocytes (Fig. 1b, c). Western blotting for TRIM16 protein was also performed from cultured keratinocytes. Deletion of the TRIM16 protein was confirmed by immunoblotting using a TRIM16 custom-made polyclonal primary antibody raised in rabbit against mouse (Biosource, CA, USA), indicating the reduced TRIM16 protein expression in heterozygous TRIM16^+/flox^ mouse and the loss of both isoforms in homozygous TRIM16^flox/flox^ mouse (Fig. 1b, c). Characterization of general features, such as food consumption, body and tail length was performed to determine differences between genotypes. Food consumption was measured by specific genotypes and the average amount of food consumed was divided between the numbers of mice in the cage. These were based on individual litters. No difference in food consumption and phenotype was observed in TRIM16 keratinocytes-specific knockout mice (Fig. 1d).

### Heterozygous TRIM16^+/flox^ mice have decreased latency of papilloma development

**Fig. 2:**
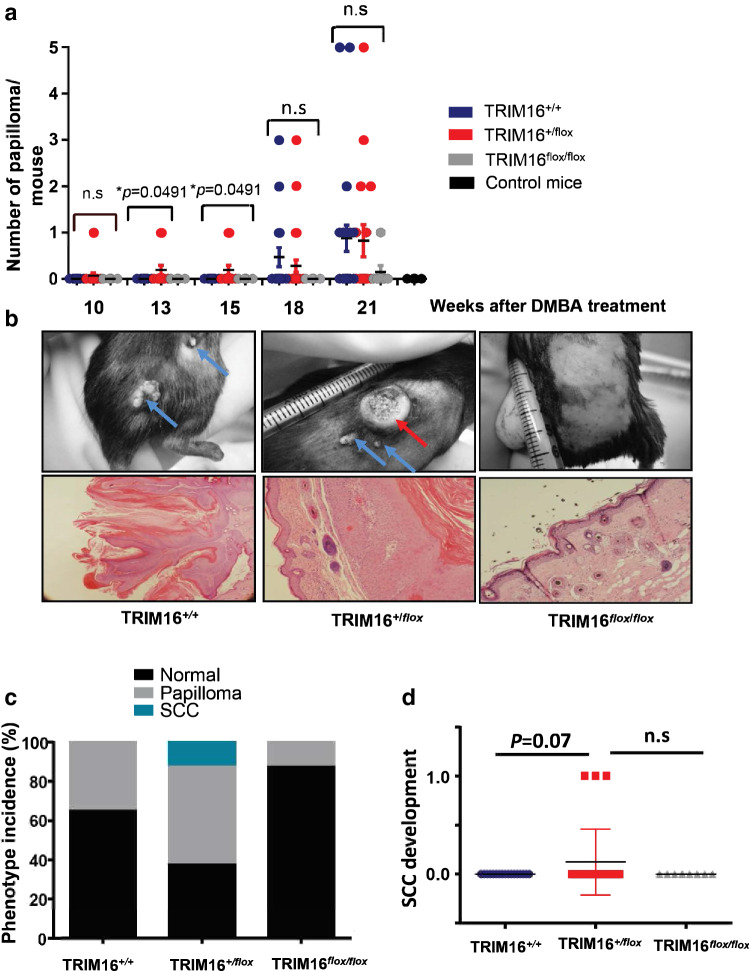
Heterozygous TRIM16^+/flox^ mice have decreased latency of papilloma development. **a** Latency of papilloma development was measured by counting the number of papilloma lesions in each cohort of mice every 3 weeks from week 10–21 following a single dose of 9,10-dimethylbenz[a]anthracene (DMBA) tumour initiation treatment. Topical tumour promoting TPA (12-O-tetradecanoylphorbol-13-acetate) treatment was administered twice weekly. The number of papilloma and mouse genotype is counted over a period of 21 weeks post-DMBA tumour initiation. An ANOVA statistical test was performed to assess significance of change in number of lesions present at each time point. **b** Representative examples are shown of papilloma (blue arrow) and squamous cell carcinoma (SCC; red arrow) with respective confirmatory histology shown below. **c** The phenotype incidence per genotype is shown with normal skin (black), papilloma (grey) and SCC (teal) as indicated. **d** The development of SCC by genotype is shown. Comparison between TRIM16^+/+^*N* = 22, TRIM16^+/flox^*N* = 33 was analysed by Student’s *t* test *P *= 0.07

To study the role of TRIM16 in skin cancer development, we used the classical two-stage skin carcinogenesis challenge method outlined by Abel and colleagues for the C57/BL6 mouse strain (Abel et al. 2009). Briefly, tumour development was initiated in mice with a single dose of DMBA (97.5 nmol), followed by twice weekly treatments of TPA (6.5 nmol). First, we assessed the loss of keratinocyte TRIM16 expression on the development of skin papilloma after carcinogen treatment, as we have previously reported the relationship between TRIM16 loss and progression from normal skin to squamous cell carcinoma (Cheung et al. 2012). We compared the number of skin papilloma observed per mouse between the different genotypes over 21 weeks (Fig. 2a). We observed a statistically significant increase in the development of papillomas in the heterozygous TRIM16^+/flox^ mice compared to other genotypes at weeks 13 and 15 (*P = 0.0491), but not at weeks 18 and 21. There was no significant difference between homozygous TRIM16^flox/flox^ mice and wild-type mice with both TRIM16 alleles intact.

Representative images of papilloma development are shown with papilloma development indicated by blue arrows (Fig. 2b). Interestingly, 3/30 TRIM16 heterozygous TRIM16^+/flox^ mice developed histologically confirmed squamous cell carcinomas (SCC, red arrow). In contrast, 0/22 TRIM16^+/+^ wild-type mice developed SCC and 0/8 TRIM16^flox/flox^ homozygous mice. The incidence by phenotype for papilloma and SCC development is shown (Fig. 2c). We found an increased incidence of papilloma in the heterozygous TRIM16^+/flox^ mice compared to wild-type and homozygous mice. The statistical analysis of SCC development between genotypes did not reach significance (*P *= 0.07) (Fig. 2d). In addition, we saw no significant difference in the size of the papilloma development between genotypes. These data indicate that partial loss of TRIM16 (in the heterozygous knockout mice) may facilitate papilloma and SCC development, but complete loss of TRIM16 (in the homozygous knockout mice) did not show increased tumorigenesis.

### TRIM16 knockout mice developed lymph node pigmentation and increased the potential for producing lymph node metastases

**Fig. 3:**
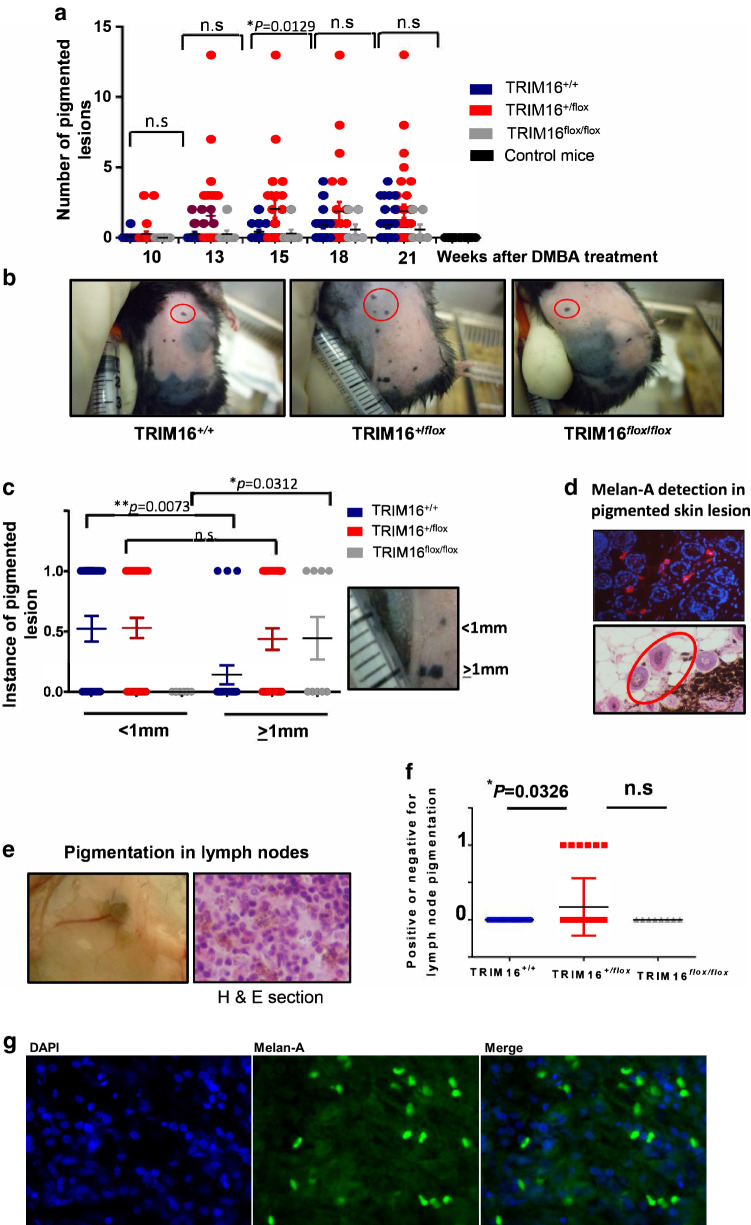
TRIM16 knockout mice developed lymph node pigmentation and increased the potential for producing lymph node metastases. **a** Latency of pigmented cell lesion development was measured by counting the number of pigmented lesions in each cohort of mice every 3 weeks from week 10–21 following a single dose of DMBA tumour initiation treatment. Topical tumour promoting TPA treatment was administered twice weekly. An ANOVA statistical test was performed to assess significance of change in number of lesions present at each time point. **b** Representative images of wild-type (TRIM16^+/+^, *N* = 22), heterozygous (TRIM16^+/flox^, *N* = 33) and homozygous (TRIM16^flox/flox^, *N* = 8) skin-specific keratinocyte knockout mice with pigmented lesions indicated by red circles. **c** Melanocytic lesion size was measured as ≥ 1 mm or < 1 mm, 0 indicates no lesion and 1 indicates a lesion. Statistical analysis was performed for each genotype with smaller or larger lesions using Student’s *t* test. **d** Immunofluorescence using Melan-A-specific antibody (red) and DAPI nuclear stain (blue) confirming the presence of Melan-A within the lesions. The proliferation of melanocytic tissue is confirmed by histological analysis using H&E staining. **e** The right inguinal lymph nodes closest to the carcinogen application site were resected for each mouse and visible pigmentation was observed, and this was confirmed by microscopic observation in haematoxylin and eosin (H&E) cross sections. **f** The presence of microscopic pigmentation between genotypes was assessed. Statistical analysis was performed for each genotype with smaller or larger lesions using Student’s *t* test *P  ≤ 0.05. **g** The sections of lymph node were probed with Melan-A antibody (Green) and a nuclear stain, DAPI (blue). Mouse Alexa fluor 488 secondary antibody was used to detect Melan-A positivity

Next, we assessed the impact of keratinocyte TRIM16 loss on the development of melanoma in the skin after carcinogen treatment. We counted the number of cutaneous melanomas per mouse over 10–21 weeks of TPA treatment (Fig. 3a, b). We noted an increase in the number of melanomas in the heterozygous KRT14-*Cre*/TRIM16^+/flox^ mice, compared to other genotypes, at 15 weeks (^*^P = 0.0129). As melanoma skin lesion size is an important prognostic indicator clinically (Abbasi et al. 2008), we compared the lesion size for the different genotypes to determine if there was a correlation with loss of keratinocyte TRIM16 expression. We categorized the lesion size as either < 1 mm (score = 0) or ≥ 1 mm (score = 1) for the three genotypes after 21 weeks of TPA treatment. We found that wild-type TRIM16^+/+^ mice developed smaller melanoma lesions with skin carcinogen treatment (**P = 0.0073) compared to either TRIM16 keratinocyte knockout genotype mice (Fig. 3c). Homozygous TRIM16^flox/flox^ knockout mice exclusively developed larger melanomas compared to other genotypes (*P = 0.0312). Heterozygous TRIM16^+/flox^ knockout mice developed both large and small lesions. We confirmed the melanocytic origin of the skin lesions using immunofluorescent detection of Melan-A (Fig. 3d, representative image is shown) (Mahmood et al. 2002). Interestingly, we observed some pigmented cells that were separated from the main melanoma lesion (Fig. 3d, red circle) and we hypothesized that these pigmented cells may be migrating and metastasizing cells.

As melanoma is a highly metastatic tumour type, we investigated the potential role of suppression of TRIM16 expression in metastasis of melanocytic lesions to the surrounding lymph nodes. To assess this, we harvested the inguinal lymph nodes that were closest to the site of lesion development for all mice (*N* = 22 for TRIM16^+/+^, *N* = 33 for TRIM16^+/flox^, *N* = 8 for TRIM16^+/+^). We visually observed pigmentation in a number of lymph nodes in the TRIM16^+/flox^ mice (6/33) that was also observed in histological cross sections of the lymph node (Fig. 3e). We analysed the difference between genotypes to determine if keratinocyte TRIM16 loss affected the incidence of metastasis. We found that only heterozygous TRIM16^+/flox^ knockout mice showed evidence of inguinal lymph node pigmentation which is concordant with the heterozygous TRIM16^+/flox^ knockout mice, developing a greater number of melanoma skin lesions and SCCs (Fig. 3f). Furthermore, the observed pigmentation in lymph nodes of heterozygous mice was Melan A-positive (Fig. 3g) by immunofluorescence, which suggests that metastatic melanoma has occurred.

## Discussion

In the human epidermis, 1 melanocyte interacts with approximately 36 keratinocytes to supply UV protective melanin (Seiberg 2001; Kippenberger et al. 1998). Furthermore, melanocytes are intricately regulated by keratinocytes and stromal factors in the skin (Santiago-Walker et al. 2009). These can be regulated by paracrine growth factors and cell–cell adhesion molecules (Haass et al. 2005). Melanocytes escape keratinocyte-regulated growth control by downregulating adhesion molecules such as E-cadherin (Haass et al. 2005), increasing melanoma-to-melanoma and melanoma-to-fibroblast cell adhesion molecule, N-cadherin and loss of cell anchorage due to changes in expression of integrin protein family members (Haass et al. 2005; Jamal and Schneider 2002). Our data demonstrated that loss of TRIM16 in a keratinocyte-specific knockout mouse model resulted in the development of larger melanocytic lesions in homozygous TRIM16 deleted mice after skin carcinogen challenge. The development of larger melanocytic lesions may indicate an increase in either radial migration of cells and/or increased melanocytic cell proliferation in TRIM16 keratinocyte knockout mice. Our result may also be due to a paracrine loss of TRIM16 signalling to melanocytes from the adjacent keratinocytes, since both TRIM16 and its target gene, IFNβ1, are known to be secreted into the extracellular environment (Munding et al. 2006b; Fujisawa et al. 1997; Kariko et al. 2004). Our own study showed that TRIM16 gene silencing reduced cell proliferation in melanocytes and melanoma cells (Sutton et al. 2014); it is possible that tumours may develop at a reduced rate in vivo, but tumours that do arise may have a more aggressive disposition due to an increase in EMT phenotype, given the increase in EMT markers, that may occur with TRIM16 silencing. This requires more study, in particular, the assessment of TRIM16 gene silencing in SCC and the evaluation of EMT markers. In addition, evaluation of EMT markers in SCC tumours comparing wild-type, heterozygous and homozygous TRIM16 mice may provide insight into the molecular pathology of tumour development in vivo.

Flower (Fwe) deficient mice have a reduced susceptibility to skin papilloma formation (Petrova et al. 2012). Like TRIM16 knockout mice, Fwe mice have no dicernable phenotype but display a significantly lower number of papilloma after DMBA/TPA carcinogen treatment compared to wild-type and heterozygous mice (Petrova et al. 2012). In the skin-specific TRIM16 heterozygous knockout mice, it is observed that a reduced latency and increased number of papilloma develop in TRIM16 heterozygous mice. However, loss of both copies of TRIM16 results in fewer papilloma. This suggests that loss of a single copy of TRIM16 increases tumour development and reduces latency, but loss of both copies abrogates this result. It is possible that a gene compensation effect is occurring by which loss of TRIM16 results in an increased expression of other genes (possibly members of the TRIM family) to compensate the loss of TRIM16. It would be interesting to test this hypothesis by performing a PCR or microarray of tissues from TRIM16 homozygous mice and determining the expression levels of TRIM family members compared to the heterozygous mice. Candidate TRIM compensation genes could be validated in vitro by knockdown of TRIM16 in SCC cells and overexpression of the candidate compensation gene to determine if TRIM16 effects on cell proliferation are ablated with gene compensation. Alternatively, the ablation of SCC development in TRIM16 homozygous knockout mice may indicate TRIM16 functions in vivo as an oncogene. An example of this is TRIM27, which exhibits complex function in cancer being characterized as both a tumour suppressor and oncogene (Hatakeyama 2011). TRIM27 regulates RARα through PML, and colocalizes with the PML-RARα fusion in acute promyelocytic leukaemia (APL). TRIM27 also translocates with the RET tyrosine kinase giving higher catalytic activity than RET alone in lymphoma, and resulting in increased cell proliferation and tumorigenesis (Hatakeyama 2011). To contrast the seemingly oncogenic activity of TRIM27, the protein has also been shown to induce apoptosis through a mechanism dependant on JUN N-terminal kinase (Dho and Kwon 2003). This profile of TRIM27 function in cancer suggests that the activity of TRIM proteins can be pleiotropic and, hence, TRIM16 may suppress cell proliferation and migration in melanoma, but has the potential to increase tumorigenesis in SCC. Further investigation is required to determine if TRIM16 is a tumour suppressor protein in melanoma and this requires developing a melanocyte-specific TRIM16 knockout mouse model that ascertains the function of TRIM16 in contributing to melanomagenesis.

In conclusion, this study suggests that keratinocytes influence melanoma development and provides the first insight into how keratinocyte TRIM16 expression impacts melanoma development. It also provides an intriguing possibility that keratinocyte TRIM16 expression may suppress melanoma cell metastasis. More detailed studies are required to determine the nature of the mechanism between TRIM16 expression and melanocytes including how exogenous TRIM16 secretion influences melanocyte cell proliferation and migration. Previously, we have shown the expression of TRIM16 to modulate IFNβ1 expression and reduce cell proliferation and migration in a manner dependent on IFNβ1 expression (Sutton et al. 2014). Exogenous treatment with both IFNα2 and IFNβ1 exerted antitumour and anti-metastatic effects, particularly to lymph nodes, in human melanoma xenografts (Roh et al. 2013). We hypothesize that keratinocyte loss of TRIM16 can increase melanoma cell migration and metastasis due to reduced paracrine expression of IFNβ1. The interplay between TRIM16 expression and innate immune response has been suggested by the observation that TRIM16 can directly bind to interleukin-1β and other components of the inflammasome (Munding et al. 2006a). Thus, reactivation of TRIM16 expression in keratinocytes may prevent metastasis from localized melanoma.
